# Retraction Note to: Quality of life in endometrial cancer survivors: single institution experience in Slovakia

**DOI:** 10.1186/s12955-021-01750-8

**Published:** 2021-04-01

**Authors:** Erik Lajtman

**Affiliations:** Gynecology and Obstetrics Department, Faculty Hospital Nitra and Constantine The Philosopher University in Nitra, Spitalska 6, 949 01 Nitra, Slovakia

## Retraction to: Health and Quality of Life Outcomes (2020) 18:221 https://doi.org/10.1186/s12955-020-01474-1

The Editor-in-Chief has retracted this article because it has been previously published by the same author [1]. This article is therefore redundant.

The author has not responded to correspondence regarding this retraction.
